# The ParlaMint corpora of parliamentary proceedings

**DOI:** 10.1007/s10579-021-09574-0

**Published:** 2022-02-02

**Authors:** Tomaž Erjavec, Maciej Ogrodniczuk, Petya Osenova, Nikola Ljubešić, Kiril Simov, Andrej Pančur, Michał Rudolf, Matyáš Kopp, Starkaður Barkarson, Steinþór Steingrímsson, Çağrı Çöltekin, Jesse de Does, Katrien Depuydt, Tommaso Agnoloni, Giulia Venturi, María Calzada Pérez, Luciana D. de Macedo, Costanza Navarretta, Giancarlo Luxardo, Matthew Coole, Paul Rayson, Vaidas Morkevičius, Tomas Krilavičius, Roberts Darǵis, Orsolya Ring, Ruben van Heusden, Maarten Marx, Darja Fišer

**Affiliations:** 1 Department of Knowledge Technologies, Jožef Stefan Institute, Ljubljana, Slovenia; 2 Institute of Computer Science, Polish Academy of Sciences, Warsaw, Poland; 3 Institute of Information and Communication Technologies, Bulgarian Academy of Sciences, and Sofia University “St. Kl. Ohridski”, Sofia, Bulgaria; 4 Department of Knowledge Technologies, Jožef Stefan Institute and Faculty of Computer Science and Informatics, University of Ljubljana, Ljubljana, Slovenia; 5 Institute of Information and Communication Technologies, Bulgarian Academy of Sciences, Sofia, Bulgaria; 6 Institute for Contemporay History, Ljubljana, Slovenia; 7 Institute of Computer Science, Polish Academy of Sciences, Warsaw, Poland; 8 Institute of Formal and Applied Linguistics, Faculty of Mathematics and Physics, Charles University, Prague, Czech Republic; 9 The Árni Magnússon Institute for Icelandic Studies, Reykjavík, Iceland; 10 University of Tübingen, Tübingen, Germany; 11 Dutch Language Institute, Hague, The Netherlands; 12 Institute of Legal Informatics and Judicial Systems CNR-IGSG, Florence, Italy; 13 Institute of Computational Linguistics CNR-ILC, Pis, Italy; 14 Universitat Jaume I, Castellón de la Plana, Spain; 15 Univ. Federal de Minas Gerais, Belo Horizonte, Brazil; 16 University of Copenhagen, Copenhagen, Denmark; 17 Univ. Paul Valéry Montpellier 3, Montpellier, France; 18 Lancaster University, Lancaster, UK; 19 Kaunas University of Technology, Kaunas, Lithuania; 20 Vytautas Magnus University, Kaunas, Lithuania; 21 University of Latvia, Riga, Latvia; 22 Centre for Social Sciences, Budapest, Hungary; 23 Universiteit van Amsterdam, Amsterdam, The Netherlands; 24 Arts Faculty, University of Ljubljana, and Institute of Contemporary History, Ljubljana, Slovenia

**Keywords:** Parliamentary proceedings, Comparable corpora, TEI

## Abstract

This paper presents the ParlaMint corpora containing transcriptions of the sessions of the 17 European national parliaments with half a billion words. The corpora are uniformly encoded, contain rich meta-data about 11 thousand speakers, and are linguistically annotated following the Universal Dependencies formalism and with named entities. Samples of the corpora and conversion scripts are available from the project’s GitHub repository, and the complete corpora are openly available via the CLARIN.SI repository for download, as well as through the NoSketch Engine and KonText concordancers and the Parlameter interface for on-line exploration and analysis.

## Introduction

The unique content, structure and language of records of parliamentary debates make them an important object of study in a wide range of disciplines in social sciences and humanities, such as political science (van Dijk, 2010), sociology (Cheng, 2015), history (Pančur and Šorn, 2016), discourse analysis (Hirst e al., 2014), sociolinguistics (Rheault et al., 2016), and multilinguality (Bayley, 2014). With an increasingly decisive role of parliaments and their rapidly changing relations with the public and the mass media on the one hand, and the executive branch and international organisations on the other, further empirical research and development of integrative analytical tools that enable comparable, trans-national analyses are necessary to achieve a better local and global understanding of parliamentary discourse as well as its wider societal impact, in particular with studies that represent diverse parts of society (women, minorities, marginalised groups), cross-cultural studies (Hughes et al., 2013), and, especially in recent times, studies of regional and global disasters, such as the COVID-19 pandemic (Neuhold, 2020).^1^

The most distinguishing characteristic of records of parliamentary debates is that they are essentially transcriptions of spoken language produced in controlled and regulated circumstances and are rich in valuable (sociodemographic) meta-data. They are also easily available under various Freedom of Information Acts set in place to enable informed participation by the public and to improve effective functioning of democratic systems, and are thus not subject to copyright or personal privacy protection, making the datasets even more valuable for researchers with heterogeneous disciplinary backgrounds. Given these reasons and the fact that parliamentary proceedings are often available on-line, many researchers have already compiled corpora of parliamentary proceedings, and there are numerous studies of parliamentary speeches.

As a case in point is the long-standing involvement of the CLARIN research infrastructure for language resources and technology, which has organised a number of events related to parliamentary data:

- CLARIN Travelling Campus “Talk of Europe” with three Creative Camps (2014, 2015), which compiled and used the proceedings of the European Parliament, curated as linked open data (van Aggelen et al., 2017);^2^
- CLARIN-PLUS cross-disciplinary workshop “Working with parliamentary records” (2017);^3^
- ParlaCLARIN LREC Workshop series on creating and using parliamentary corpora (2018 and 2020) (Fišer et al., 2018, 2020);
- CLARIN Resource Families (Fišer et al., 2018): “Parliamentary corpora” (2018–2019) (Fišer and Lenardič, 2018);^4^
- CLARIN ParlaFormat workshop (2019).^5^

Of these, the ParlaFormat workshop, organised by the CLARIN Interoperability Committee attempted to address a problem with all existing national parliamentary corpora, namely, that they are encoded in many different ways and contain different information, presenting a barrier to their interchange, re-use and comparison. The result of the workshop and follow-up activities were the Parla-CLARIN recommendations for encoding parliamentary corpora (Erjavec and Pančur, 2019),^6^ which give guidelines for good practice, and define a schema based on the Text Encoding Initiative Guidelines (TEI, 2017).^7^

In the process of developing the recommendations, the second version of the siParl corpus (Pančur and Erjavec, 2020; Pančur et al. 2020) was also produced. siParl is a carefully encoded and automatically linguistically annotated collection of parliamentary debates from the Assembly of the Republic of Slovenia from 1990 to 2018, with over 1 million speeches and 200 million words. This was the first substantial corpus to be fully Parla-CLARIN encoded, where the encoding was also informed by the experiences from tutorial development on how corpora can be used to investigate language use and communication practices in the context of political discourse (Fišer and Pahor, 2020) which relied on siParl 1.0 (Pančur et al., 2019) as its empirical basis. The work on this tutorial revealed various shortcomings of the first version, which were addressed in the encoding and structure of siParl 2.0^8^ and the development of the Parla-CLARIN recommendations.

Taking the Parla-CLARIN encoding as its basis, the ParlaMint project was the next step in the CLARIN-supported development of interchangeable and interoperable comparable corpora of parliamentary transcriptions, and its results are presented in this paper, which is structured as follows: Sect. 2 details the developed corpora, including their compilation, composition and size; Sect. 3 illustrates the Parla-CLARIN-based ParlaMint encoding of the corpora; Sect. 4 overviews access to the corpora for on-line analysis and download; and Sect. 5 gives some conclusions and plans for future work.

## Compilation and overview of the corpora

The ParlaMint project^9^ lasted from November 2019 to May 2021, and was divided into two stages: first (from November 2019 to September 2020), the core partners developed and tested the workflow on their corpora (Croatian, Bulgarian, Polish, Slovene), after which a call was launched inviting authors of additional corpora of national parliaments from Europe to join the initiative, with a large number (13) of responses; a special case was the Spanish corpus, which joined the project later on a voluntary basis. The additional corpora were to follow the same meta-data and data requirements, encoding schema and linguistic annotation as the first four. The main goal was to produce a uniformly encoded, comparable, and linguistically annotated set of corpora of the participants’ countries, which were to be focused (according to the initial showcase research question) on the COVID pandemic. To this end, the corpora were centred around November 2019, with speeches after this date taken to belong to the COVID subcorpus, and those before to the reference subcorpus. In all cases, the assumption was that the individual partners had already compiled their parliamentary corpus in the required time-frame or could access the digital source for their corpus and then convert and annotate it according to the ParlaMint requirements.

The ParlaMint encoding follows the already mentioned Parla-CLARIN recommendation, but was, already in the first stage, significantly constrained to make the corpora maximally interoperable, so that the same scripts could be used to convert the corpora into various derived formats or to analyse their contents. For this, a set of bespoke (but Parla-CLARIN compatible) RelaxNG schemas were written that were later also supplemented with XSLT scripts to validate the corpora; good validation was crucial for the interoperability of the corpora, especially as the corpora were not produced centrally, but by a different partner each.

In practice, the development of the final encoding was very much a cyclic process, with new corpora containing new phenomena to be encoded, at times also leading to revisions of already accepted encoding practices, and hence to revisions of previously completed corpora. For the project-final version of the corpora, it even became advantageous to introduce an XSLT script to process all the corpora and unify certain aspects of the encoding. The exemplification of the ParlaMint encoding is discussed in detail in Sect. 3.

The meta-data required for every ParlaMint corpus followed from our previous experiences gained in developing the already mentioned tutorial. There it turned out that e.g. it is crucial to distinguish Members of Parliament (MPs) from guest speakers (of which there are many but have few speeches) and regular speakers from the sessions chairs (who give many speeches, but mostly on procedural matters), as otherwise various comparative analyses give skewed and uninformative results. In addition to the required meta-data, additional available meta-data was also included in the ParlaMint corpora, but its types and coverage are dependent on individual corpora.

The corpora were prepared (and are distributed) in two variants: the first is the fully marked-up corpora, but with plain text of the speeches, while the second is identical to the first, but with added linguistic annotations to the texts of the speeches. It was up to the partners to select the tool for the analysis of their language, but the requirement was that the automatic linguistic mark-up should contain tokenisation, sentence segmentation, part-of-speech tags, morphological features, and syntactic analyses, all according to the Universal Dependencies formalism (de Marneffe et al., 2021) for the particular language. Furthermore, we required named entity (NE) annotations, using the standard PER, LOC, ORG, and MISC classes.

Important for the corpus compilation workflow was the use of Git, in particular GitHub,^10^ as it provided a distribution medium for the latest schemas, scripts and, in lieu of comprehensive documentation on the precise format, samples of individual corpora.^11^ GitHub also supports reporting, tracking and archiving issues, which were expected to be the main communication channel regarding the development of the corpora. However, only some partners embraced this, while others preferred to use email, a much more labour-intensive way of resolving issues. Nevertheless, there were 58 reported and closed issues during the project lifetime, along with 530 commits.

So far, the ParlaMint corpora have been published in three versions. Version 1.0 contained the initial four corpora with the main intention to serve as a model for what needed to be done by the new partners. Version 2.0 was released towards the end of the project as the near-final version of the corpora. While it was still missing some languages, the main reason for this release was to enable the ParlaMint data to be used in the scope of the Helsinki Digital Humanities Hackathon^12^ which took place in May 2021. The experience of the participants was very welcome, and led to changes of several aspects of the final released version 2.1 of the project.

In the rest of this section, we first describe the corpus compilation process from the perspective of the individual corpora, and then give a quantitative overview of the ParlaMint corpora over their most important dimensions, including their basic characteristics, speaker metadata, the speeches, and associated mark-up.

### Compilation of individual corpora

The greatest amount of work for the individual partners was to prepare their corpora, and this section details the source, up-conversion to the ParlaMint format and the tools used for linguistic annotation of each corpus.

#### Belgium

The data from the federal parliament were obtained by scraping the source files in HTML (apparently exported from Microsoft Word) from the parliamentary website^13^, and contain speeches from November 2015 to August 2020.

The conversion to the ParlaMint format consisted of several steps to transform and enrich the source data, using XSLT and Python scripts. The main challenges were related to the unstructured nature of the source data, which made it nontrivial to recognise the beginning and ending of the speeches and to separate them into monolingual segments.

The linguistic processing of the corpus was complicated by the fact that the corpus is bi-lingual, with the language changing even inside the same speech, however, being uniform inside one paragraph. The linguistic annotation proceeded as follows: 1. Dutch/French language identification on the level of paragraphs, using a combination of the Microsoft Office language identification present in the source documents and the Python language identification module langdetect;^14^ 2. tokenisation (Dutch and French) and tagging/lemmatisation (Dutch only, tagging and lemmatisation for French relies on the UD-pipe) by means of the INT in-house tagger based on Support Vector Machines; and 3. dependency parsing and NER, using the Trankit (Nguyen et al., 2021) universal dependencies pipeline.

#### Bulgaria

The gathering and processing of Bulgarian parliamentary data started already in 2010 (Osenova and Simov, 2012) and the first version of the resulting corpus (50 million words, 2006–2012) was made available through a concordance service.^15^ Here, the data was converted into XML with very basic TEI elements, such as speakers and speeches without further refinements.

For ParlaMint, all the publicly available plenary sessions (October 2014 to July 2020) were manually downloaded in HTML format from the official website of the Bulgarian National Assembly.^16^ The necessary metadata (such as information about the MPs and their roles, parties, etc.) was partially present on the Parliament website, with the rest gathered from Wikipedia and other internet sources. The conversion from HTML was performed in an incremental way: first, it was saved in a basic XML format and then converted and validated to be compliant with the ParlaMint schema.

The linguistic annotation of the corpus was performed automatically by the CLASSLA pipeline (Ljubešić and Dobrovoljc, 2019),^17^ a fork of the well-known Stanford Stanza pipeline (Qi et al., 2020).^18^ The reason for preferring the CLASSLA pipeline over Stanza is that CLASSLA models are based on a larger training dataset, use large inflectional lexicons, and have support for Named Entity Recognition (NER). The same holds for Croatian and Slovenian, for which CLASSLA was also used to perform linguistic annotation.

The performance of linguistic annotation is similar for Bulgarian, Croatian and Slovenian: task-based F1 score^19^ of morphosyntactic tagging lies between 94% and 97%, of lemmatisation between 98% and 99%, and of dependency parsing between 87% and 94%. The NER module of the tool has not been evaluated yet.

It should be noted that the accuracy scores reported for Bulgarian, as well as for all the other country corpora were calculated on reference corpora, so their performance on the parliamentary data would be different, and most likely somewhat lower.

#### Croatia

The source data for ParlaMint-HR was a JSON dump of the parliamentary data of the Croatian ParlaMetar platform,^20^ and the speeches and speaker metadata of the 9th term (November 2016 to May 2020) of the (unicameral) Croatian parliament. Unfortunately, the dump did not contain the exact dates of the speeches, but only their session number, of which there are 16, so this corpus, unlike all the others, gives only a roughly 3-month interval for each component file (and hence its speeches), rather than the exact date, as is the case for all other corpora.

To up-convert JSON to ParlaMint XML, a Python script was used for the basic conversion, and a Perl heuristic to identify and mark-up transcriber comments.

The automatic linguistic annotation was performed by the CLASSLA pipeline (Ljubešić and Dobrovoljc, 2019), using the standard-language Croatian models, and yielding morphosyntactic tagging, dependency parsing and NER. As mentioned above, F1 of morphosyntactic tagging is 94–97%, F1 of lemmatisation 98–99%, and F1 of dependency parsing 87–94%.

#### Czech Republic

The Czech team started compiling Czech parliamentary data into ParCzech corpora very recently (Hladká et al., 2020). The first ParCzech corpus PS7 1.0 was compiled before the ParlaMint project started and was published in a TEI-inspired format (Hladká et al., 2020). The ParlaMint project had considerable influence on the ParCzech corpora, and the latest version of ParCzech 3.0 (Kopp et al., 2021, 2021) follows the ParlaMint schema and slightly extends it with respect to the alignment of transcriptions and audio recordings.

The ParlaMint-CZ corpus contains all Czech Chamber of Deputies speeches from November 2013 to April 2021. It preserves as many source features as was possible while not making changes to the ParlaMint schema. Including the original page breaks in ParlaMint-CZ allows storing the hypertext links to the source web pages and their audio file links.

In addition to the standard 4-class ParlaMint NE classification used in the ParlaMint corpora, a more detailed Czech-specific NE taxonomy (Straková et al., 2017) is used in ParlaMint-CZ. This taxonomy distinguishes 46 hierarchically organised NE classes, which can be nested, and 4 container NEs (e.g., first name and surname form a complex personal name NE). This rich NE annotation was performed with NameTag 2 (Straková et al., 2019)^21^ with czech-cnec2.0-200831 model^22^ that reaches F1 score 83.44% for 46 two-character types and 87.04% for 6 one-character supertypes on the CNEC2.0 test data.

UDPipe 2 (Straka, 2018)^23^ with the pdt-ud-2.6-200830 model^24^ was used to tokenise and morphologically and syntactically annotate ParlaMint-CZ. Raw-text F1 model scores for the UD2.6 data are 97.13% for morphosyntactic tagging and 99.09% for lemmatisation. Labeled Attachment Score is 92.03%, and Morphology-Aware Labeled Attachment Score is 87.79%.

All linguistic annotations have been performed automatically and then slightly improved with rule-based interventions that patched annotation failures, e.g. root relations on nodes in the middle of dependency trees.

#### Denmark

The Danish parliament speeches are accessible online in XML format from the ftp server of the Danish Parliament.^25^ The speeches in ParlaMint-DK partly overlap with speeches in The Danish Parliament Corpus 2009–2017, v.1, which has been available from CLARIN-DK since 2018.^26^ The speeches from the latest parliamentary year (2020) were not published as the final edition when they were downloaded for the ParlaMint project and they are therefore coded with a “preliminary version” mark in the subtitles.

A ParlaMint specific workflow was built in which the speeches were converted to the common ParlaMint format and segmented taking into account the structure of the speeches.

The linguistic annotations were produced with the Text Tonsorium workflow management system, which can be run through CLARIN-DK.^27^ It uses a combination of CST-tools and dapipe,^28^ a tool that calls UDPipe for Danish. Some of the tools in the Text Tonsorium system were adapted in order to deal with the parliament speeches (Jongejan et al., 2021).

The F1-score over standard test data is 99% for segmentation, 94% for morphosyntactic annotation, 98% for lemmatisation, and 98% for NER.^29^

#### France

The proceedings of the debates in the plenary sitting of the Assemblée Nationale are published in an XML format since May 2013.^30^ The Web site also provides a number of datasets (in XML or JSON formats) describing the metadata used for the proceedings and structured according to three main categories: actors (MPs or guest speakers), mandates and bodies (acteurs, mandats, organes).^31^

The source XML files were converted into the ParlaMint format in several steps by a set of scripts in Python, Perl and XSLT. The pipeline generates the corpus based on initial and end sitting dates.

The linguistic annotation was automated by means of a Python script combining an XML parser module with the Stanza NLP pipeline using the French models ud-french-gsd-2.7 for UD and aij-wikiner-fr for NER. Performance tests for French give F1 scores of 96% for morphosyntactic tagging, 98% for lemmatisation and 89% for Labeled Attachment Score (LAS). The F1 score of Stanza’s pretrained NER model is assessed at 93%.

The code base for ParlaMint was derived from a development initiated in 2018 and called TAPS (Transcription and Annotation of Parliamentary Speech), which is still supported (Diwersy and Luxardo, 2020) and allows processing the French data with additional platforms besides those mentioned in Sect. 4.1.

#### Hungary

The Hungarian parliamentary corpus contains two types of speeches (interpellations and urgent questions) from plenary sessions of the National Assembly of Hungary, terms 7 and 8 (May 2014 – December 2020). The source data was downloaded from the official page of the Hungarian National Assembly^32^ and converted to the ParlaMint format with dedicated scripts.

To create the linguistically annotated files, we had to use three different linguistic tools, since none of the available NLP tools for Hungarian is capable of providing all the required analyses. For morphosyntactic annotation, we used an older version of the magyarlanc linguistic toolkit,^33^ with the tool achieving 96.3% accuracy on its test dataset. The syntactic analysis was performed by UDPipe (Straka and Straková, 2017), which, for Hungarian, achieves 70.4% BLEX (bi-lexical dependency) Score for lemmatization and 78.5% Labeled Attachment Score (LAS) for dependency parsing. NER was done by the tool created by the MTA-SZTE Research Group on Artificial Intelligence, which reportedly achieves 94.8% F1.^34^ The output of the three tools was merged with a Java program, where the baseline of the merging procedure were the text files tokenised by UDPipe. On the basis of this analysed version, JavaScript was used to add the appropriate XML tags, including the required metadata.

#### Iceland

The gathering of the Icelandic parliamentary data started in 2017 and was first published as a part of the Icelandic Gigaword corpus (Steingrímsson et al., 2018) in 2018. In 2020 the corpus was published individually under the name of IGC-Parl (Steingrímsson et al., 2020) and mostly followed the Parla-CLARIN recommendation. IGC-Parl contains speeches from 1911 to 2019 while the speeches in ParlaMint date from January 2015 to the end of September 2020. All the speeches were downloaded as HTML from the website of the Icelandic parliament Althingi.^35^ The metadata was saved in database and the transcriptions in text files. The XML files were then converted from this material, using Python scripts written for that task.

The tokenisation was performed using Tokenizer^36^ and the text lemmatised with Nefnir (Ingólfsdóttir et al., 2019). The linguistic annotation of the Icelandic corpus was twofold. The Icelandic implementation of UD-pipe was used for the universal PoS tags, and ABLTagger (Steingrímsson et al., 2019) for the morphological tagging commonly used when tagging Icelandic texts. A BERT-based model^37^ was used for NER.

#### Italy

The Italian corpus consists of all transcripts of the plenary sessions of the Senate, i.e. the assembly of the upper house of the Italian Parliament. The transcripts range from the beginning of the 17th legislative term (March 2013) to the date of corpus collection in the 18th legislative term (November 2020). Even though the transcripts are freely available through the website of the Senate in HTML format, the Information Technology Service of the Senate made them available in bulk for the considered periods. It should be noted that since 2018 the transcripts are also published in the Akoma Ntoso XML format, but in order to uniformly cover the whole time-frame (including years before 2018) the HTML format was chosen as the source format for the whole corpus. Proprietary XML tags contained in the HTML annotation were automatically converted by a Java script developed for this purpose. The script also integrates metadata of the speakers and political groups obtained from the Italian Senate Open Data access point.^38^

The automatic linguistic annotation was performed using the Italian model italianisdt-ud-2.5 of the Stanza pipeline, while the annotation of Named Entities was carried out by the ItaliaNLP NER module (Dell’Orletta et al., 2014). They both achieve state-of-the-art performances for Italian on official test sets. Namely, F1 of morphosyntactic tagging is 97.11%, of lemmatisation is 98.1% and Labeled Attachment Score (LAS) of dependency parsing is 90.84%. NER overall accuracy is 97.02%. The outputs of the linguistic and NE annotation were then automatically aligned in order to have a unified annotation. For this last step, we defined a number of alignment rules specifically devoted to handling mismatches resulting from different tokenisation approaches followed by the Stanza pipeline and the ItaliaNLP NER module.

#### Latvia

The source data for the ParlaMint-LV corpus was crawled from the Saeima’s website^39^ where verbatim reports of all the sessions of the Saeima are published in HTML format. For the ParlaMint corpus we crawled the time span from November 2012 to November 2020.

The texts were processed using a semi-automatic pipeline to identify the boundaries of speeches and speakers. The texts were then split into utterances, where each utterance contains a speech from only one speaker. The result of this processing consists of JSON files, where each file represents one session. The processed JSON files were encoded into the ParlaMint TEI format using a custom Python script. The biggest challenge was mapping the utterances to a unique speaker identifier, because speakers in the session are noted by initials and affiliation. The initials alone are not unique and the affiliations are not consistent, for example in some cases the full name is used, in other cases different abbreviations. On rare occasions even the initials and affiliation pair is not unique.

The linguistic annotation was generated using the Latvian NLP Tool Pipe-line (Znotiņš and Clrule, 2018),^40^ which provides all the necessary layers – tokenisation, morphological annotations, syntactical annotations and NER.

#### Lithuania

The collection of Lithuanian parliamentary debates transcripts and other relevant information was compiled in 2020 by the researchers working in the PolAFra project.^41^ All the transcripts of the Seimas floor debates were automatically collected from the official document search site of the Seimas.^42^ The metadata about MPs was collected from the open data portal of the Seimas,^43^ the main Seimas web portal^44^ and other sources.

The corpus was converted into the ParlaMint format using custom Python scripts that read the input transcripts and metadata about the MPs and transformed them into the required XML structures. The corpus documents were encoded by parsing the input XML documents into a DOM, traversing the documents, and applying the appropriate transformations from source to target elements. The speaker identifiers were mapped to the corpus identifiers and kept consistent.

The linguistic processing was implemented by means of a Python script combining an XML parser module within the spaCy package.^45^ The annotation pipeline included tokenisation, sentence segmentation, lemmatisation, UD part-of-speech and morphological tagging, UD dependency parsing and NER.

#### The Netherlands

In the Netherlands, the proceedings of debates of the Upper House and the Lower House are publicly available in XML format through their website.^46^ Because the metadata of the speakers is not included in these XML files, the metadata was scraped from several websites, including Wikipedia, and matched to the actors named in the original XML files. After adding the metadata, XSLT scripts were used to convert the structure of the scraped XML documents to the ParlaMint format.

For the linguistic annotation, the Trankit package (Nguyen et al., 2021) was used, a lightweight NLP package based on Transformers, usable for UD Parsing and NER, trained on a multitude of languages, including Dutch. For the task of NER, a set of training examples was manually annotated to evaluate the performance of the model on the Dutch parliamentary proceedings, where an average F1 score of 85% was achieved on the Named Entities in the parliamentary debates.

#### Poland

The data and linguistic annotation for ParlaMint-PL was taken from the Polish Parliamentary Corpus (Ogrodniczuk, 2012, 2018; Ogrodniczuk and Nitoń, 2020).^47^ The ParlaMint-PL corpus contains the stenographic record of plenary sittings of the Sejm, the lower chamber of the parliament of the Republic of Poland (8th and 9th term of office) and Senate, the upper chamber (9th and 10th term of office).

The data was converted from its TEI P5 XML representation which follows the format of the National Corpus of Polish (Przepiórkowski et al., 2012) to the ParlaMint format using Python scripts. Some errors in the original corpus were automatically corrected during conversion. Heuristics were used to convert event descriptions and comments into ParlaMint types, mostly based on typical phrases used in the text. Metadata of the MPs was retrieved from the websites of Sejm and Senate.^48^

The linguistic annotation was created automatically with Morfeusz2^49^ (utte-rance-level segmentation, tokenisation and lemmatisation, all tasks with F1 score over 99%) (Kieraś and Woliński, 2017), Concraft2^50^ (disambiguated morphosyntactic description, F1 92%) (Waszczuk et al., 2018), Liner2^51^ (named entities, F1 81%) (Marcińczuk et al., 2013) and COMBO parser^52^ (dependency structures, LAS F1 95%) (Rybak and Wróblewska, 2018).

#### Slovenia

Slovenia has a long tradition in compiling parliamentary corpora, with the already mentioned siParl 2.0 corpus (Pančur and Erjavec, 2020; Pančur et al. 2020) covering 1990–2018 being the latest release at the time when the ParlaMint project was started. The corpus contains the complete debates of National Assembly of Slovenia and was already encoded according to the Parla-CLARIN recommendations.

The siParl 2.0 corpus served as the basis for the ParlaMint-SI corpus. It was first extended to July 2020 (including the addition of speaker metadata), and the debates older than August 2014 were removed. We then converted its encoding to ParlaMint, which was performed by an XSLT script.

For the automatic linguistic annotation, as is with Bulgarian and Croatian data, we used the CLASSLA pipeline (Ljubešić and Dobrovoljc, 2019) with standard-language Slovenian models. As previously mentioned, F1 of morphosyntactic tagging is 94–97%, of lemmatisation 98–99%, and of dependency parsing 87–94%.

#### Spain

The plenary speeches of the Spanish parliament were downloaded from the Congreso de los Diputados’s website^53^ and processed by the European Comparable and Parallel Corpora (ECPC) research group, which has compiled an archive of the speeches at the Universitat Jaume I, Spain (Calzada Perez, 2017).^54^ This complete archive is an XML annotated collection of speeches from three European chambers: the European Parliament, the British House of Commons, and the Spanish Congreso de los Diputados (CD). The CD part contains speeches from 2004 to 2014 and was, as part of the ParlaMint project, expanded with speeches from 2015 to 2020.

The up-conversion of the CD speeches to EPCP XML was performed by using Perl, Python and Bash scripts, and this was subsequently converted to ParlaMint XML with XSLT scripts. Unfortunately, the original does not contain names of the chairs of the sessions, so those speeches do not have an associated speaker.

The linguistic annotation was performed using Stanza (Qi et al., 2020),^55^ with the default AnCora model for Spanish.

#### Turkey

The data for ParlaMint-TR was scraped from the official web page of the National Assembly of Turkey,^56^ where we gathered transcripts from April 2009 and to February 2021. These parliamentary transcripts are available as HTML files and were processed by custom Python scripts to extract speeches and speaker information. The speaker metadata was obtained from Wikipedia and the official parliament web page.

Conversion to the ParlaMint format was performed using custom scripts, which are, along with the manually corrected metadata, available on GitHub.^57^

Tokenisation, sentence segmentation, morphological tagging and disambiguation were performed using TRmorph (Çöltekin, 2010, 2014), syntactic annotation using UDPipe (Straka, 2018) and a freely available tool for Turkish NER.^58^ The tagging and disambiguation accuracy of TRmorph is reported to be 96% (as opposed to only 86.1% for tagging of Turkish with UDPipe), while the labeled attachment score of UDPipe for Turkish is between 55.1% and 61.4%, with raw-text input and gold-standard tags.

#### United Kingdom

The UK parliamentary corpus data from 2015 to March 2021 was gathered using the UK Parliament’s Hansard API.^59^ This provided access to speeches from the House Commons and Lords in XML format and metadata on speakers and parties. This metadata was further enhanced using the Parliamentary Open Data API^60^ which supplied additional speaker information (portraits, contact details and social media handles) in RDF format.

The XML transcriptions of speeches and metadata were combined and converted into the ParlaMint format using XSLT.

Extension functions were used to automatically annotate the data using the Stanford CoreNLP^61^ pipeline, which has, on reference treebanks, F1 90.91% on part-of-speech and 87.59% on morphological features (Qi et al., 2019).

### Overview of the corpora

ParlaMint version 2.1 contains 17 corpora with 16 main languages comprising over 22 thousand files, 5 million speeches and almost half a billion words; Table 1 gives a quantitative overview of some basic characteristics of the individual corpora.

**Table 1:** Basic information about the ParlaMint corpora including the corpus ID, the covered language(s), the houses and number of terms included, from and to months of included transcriptions, the number of years covered, the number of millions of words per year and in total

ID	Lang	Houses	Ts	From	To	Yrs	Mw/Yr	Mw
BE	nl+fr	Lower	2	2015–11	2020–08	4.8	6.50	31.37
BG	bg	Unicameral	2	2014–10	2020–07	5.8	3.42	20.02
CZ	cs	Lower	2	2013–11	2021–04	7.5	3.03	22.56
DK	da	Unicameral	–	2014–10	2020–09	6.1	4.85	29.40
ES	es	Lower	5	2015–01	2020–12	6.0	2.19	13.10
FR	fr	Lower	1	2017–07	2020–07	3.0	10.75	32.73
GB	en	Lower+Upper	4	2015–01	2021–03	6.3	17.25	109.30
HR	hr	Unicameral	1	2016–11	2020–05	3.6	5.81	20.65
HU	hu	Unicameral	2	2014–05	2020–12	6.7	0.13	0.87
IS	is	Unicameral	3	2015–01	2020–09	5.8	4.06	23.66
IT	it	Upper	2	2013–03	2020–11	7.8	3.46	26.94
LT	lt	Unicameral	2	2012–11	2020–11	8.1	1.82	14.78
LV	lv	Unicameral	2	2014–11	2021–02	6.3	1.02	6.48
NL	nl	Lower+Upper	5	2014–04	2020–11	6.6	7.74	51.45
PL	pl	Lower+Upper	4	2015–11	2020–08	4.9	5.66	27.45
SI	sl	Lower	2	2014–08	2020–07	6.0	3.34	20.19
TR	tr	Unicameral	4	2009–04	2021–02	12.0	3.65	43.99

The first column gives the country codes of the corpora, which follow ISO 3166 “Codes for the representation of names of countries and their subdivisions”. These ISO 3166-1 two-letter codes are consistently used in the ParlaMint corpora e.g. in file naming and XML identifiers. They also allow for a straight-forward extension to the European (EU) and regional parliaments (e.g. ES-CT for Catalonia), the latter covered by ISO 3166-2 “Country subdivision code” part of the standard. The second column gives the ISO 639-1 code of the main language(s) used in the corpus. All the corpora are monolingual, except for Belgium, which contains a mixture of Dutch and French. The third column gives the type of parliament of the country, in cases of bicameral parliaments specifying the transcriptions of which house are included in ParlaMint.

The next three columns give time-related information on the corpora, starting with the number of possibly partial government (so, lower house, when both are present) terms of the included speeches. These largely reflect the time-frame of the corpus, but also indicate the dynamics of (extraordinary) elections. Danish (DK) does not have information about the terms, but only sessions, so its cell is left blank. The From and To dates and hence the number of years of included speeches varies considerably, but with most corpora starting in or before 2015 and ending mid-2020 or later. With the exception of France and Croatia all corpora have more than 4.5 years worth of speeches, with Turkey having as much as 12 years.

Finally, the last two columns give the size of each corpus in words per year and as a whole. By far the largest corpus, both per year and in total, is that of Great Britain, with even the fact that it contains the speeches of both the House of Lords and of the House of Commons not fully explaining its size, but must be (as it is with the French) a result of longer or more sessions of their parliaments. In the opposite direction, the outlier is the Hungarian corpus, where its small size is due to the fact that it contains only interpellations and urgent questions from plenary sessions of the parliament.

### Metadata on speakers

The ParlaMint corpora contain significant metadata about its 11,412 speakers, which allows for various political or sociological but also linguistic studies for which speaker-related variables are required. Table 2 gives an overview of speaker-related data over the individual corpora.

**Table 2:** Metadata on speakers with the number of political parties and groups, other “organisations”, number of defined persons, those with assigned gender, how many are MPs, and how many have party affiliation(s), known date of birth, one or more associated URLs and link to their photo

ID	Prts	C/O	Orgs	Prsns	Gender	MP	Affill	Birth	URL	IMG
BE	63	10	2	775	548	548	548	548	0	548
BG	14	4	5	606	606	420	310	534	99	0
CZ	61	5	851	485	461	366	366	403	463	364
DK	19	4	2	454	454	446	454	454	0	0
ES	50	10	2	814	814	764	758	793	0	0
FR	16	0	100	670	670	609	585	664	0	0
GB	31	5	2	1901	1901	1865	1897	0	1901	1029
HR	16	2	2	322	322	182	186	168	0	0
HU	10	0	2	194	194	194	194	192	0	0
IS	10	6	2	205	205	113	201	205	0	0
IT	42	22	2	739	739	689	589	739	0	0
LT	13	20	214	799	799	247	233	247	0	0
LV	11	0	2	219	219	174	174	0	0	0
NL	29	12	3	492	492	454	457	0	0	0
PL	10	3	1	1123	1122	743	709	742	0	0
SI	15	8	5	377	377	167	163	193	78	0
TR	19	3	2	1236	1236	1223	1203	0	0	0

The first three numeric columns give the numbers related to political parties^62^ and other “organisations” that are listed in the corpora. Each is given an ID (to which information on speakers refers to), its full and abbreviated names, and, depending on the corpus, also the dates of its existence. For most of the corpora, time-stamped coalitions and oppositions of political parties are also encoded, and the number of such groupings is given in the C/O column. The “Orgs” column gives the number of organisations which are not political parties or groups. The corpora typically give only information about the one (unicameral) and two (bicameral) houses of the respective parliaments, but with three corpora, most strikingly the Czech one, encoding also information about various committees, commissions, delegations, etc. with the speakers that are their members linked to them.

The following columns give the numbers related to the defined persons,^63^ the number of which goes from 194 for Hungarian and up to 1,901 for the United Kingdom. The next column shows that all corpora have all or almost all speakers marked for their gender, where we use the traditional male/female distinction. The MP column gives the number of speakers that are marked as members of parliament; these contribute by far the most speeches in the corpora. The “Affil” column gives the numbers of speakers that are affiliated with a political party or group and which typically cover most of the MPs. Most corpora also give for most of the MPs their date of birth, which enables age-correlated studies of speeches. Finally, the last two columns give the numbers of speakers with additional information, in particular, if they have associated with them one or more URLs (Wikipedia page, official government Web page, Twitter and Facebook account). Only four corpora have this information, with by far the most exhaustive links given in the corpus of the United Kingdom. A similar situation, but with only three corpora, contains links to photos of the MPs.

### Speeches and associated mark-up

The ParlaMint corpora contain 4,927,003 speeches and 1,800,340 elements with related information. The first numeric column in Table 3 gives the number of speeches per corpus, with Hungary having just over 3000, but Turkey containing over 1.5 million. For investigations that take into account characteristics of the speakers, it is important to note how many speeches are marked with their speakers, which is, as given in the “With Speakers” column, for most corpora (almost) all of them, except for Spanish, French and Turkish, where 44.3%, 3.3% and 4.6 % are missing, respectively. In the Spanish case, this is almost exclusively due to the chairs of the sessions not being identified by name. Also important are the numbers of speeches spoken by non-chairs of the session, which is given in the next column. The proportion of such speeches varies widely between countries, although most being about half of all the speeches. Seven countries (Belgium, France, United Kingdom, Iceland, Italy, The Netherlands, Turkey) have many more, with a special case being Hungary, which has no speeches made by chairs. The last column gives the number of speeches by MPs, rather than guest speakers. Here the proportions are typically comparable with all over 90%, except for Bulgarian with 82.6%. Again, the outlier is Hungary, with 100% MP speeches.

**Table 3:** Overview of selected data from ParlaMint corpora with the number of speeches, of speeches with a known speaker, of those not spoken by the chair of the sessions, spoken by MPs, and the number of marked-up headings, notes, and incidents

ID	Speeches	W.Spks	W.NCs	W.MPs	Heads	Notes	Incidents
BE	148,425	147,940	116,214	141,340	0	140,512	865
BG	146,351	146,295	73,981	120,780	0	0	34,313
CZ	154,460	154,460	72,301	150,957	0	188,563	25,692
DK	287,144	287,144	137,210	277,835	10,544	10,544	0
ES	49,919	27,812	21,414	27,709	1728	46,965	0
FR	481,603	465,590	421,241	437,965	12,498	12,498	62,709
GB	552,103	549,710	537,928	547,305	31,389	165,648	0
HR	124,496	124,486	62,128	116,716	16	9	11,842
HU	3086	3086	3086	3086	0	2958	3752
IS	74,132	74,132	71,693	71,900	0	99	41,405
IT	79,373	79,373	50,735	78,269	11,585	192,855	61,607
LT	244,835	244,835	126,488	229,980	1752	35,406	30,155
LV	122,136	122,136	60,663	117,899	0	122,136	0
NL	474,964	474,964	351,789	463,629	0	191,113	0
PL	331,044	331,044	226,046	302,965	516	9453	112,786
SI	75,122	75,122	37,216	70,609	1240	85,111	2337
TR	692,161	660,239	432,618	660,239	0	142,415	0

The following columns quantifies the elements that appear in the corpus texts apart from speeches. Namely, the transcripts also contain session or agenda titles, names of speakers or chairs etc., which have been preserved in about half of the corpora and marked up as headings, the number of which is shown in the “Head” column. Furthermore, the transcripts contain many transcriber notes, i.e. remarks about time, voting, interruptions, applause, the fact that the speaker could not be understood etc. Such commentary was identified and marked up in the corpora in two ways. The default was to mark them up as notes, while some corpora also use more precise elements, the sum of which is shown in the “Incidents” column; these elements are “vocal” (non-lexical vocalised phenomena, e.g. exclamations from the auditorium), “kinesic” (non-vocalised communicative phenomena, e.g. applause) and “incident” (non-communicative phenomena, e.g. coughing).

## Corpus encoding and structure

As mentioned in Sect. 1, the encoding of ParlaMint corpora follows the ParlaMint schema, which is also compatible with the Parla-CLARIN recommendations. A ParlaMint corpus is composed of the root file named ParlaMint-XX (with XX being the country code), with teiCorpus being the top level element, which then contains the corpus teiHeader with corpus-wide metadata and XInclude elements gathering the files of the corpus components. The corpus components typically contain a day’s-worth of transcriptions, but this differs among the corpora, some having more files per day (corresponding to different meetings) and the Croatian one, as already explained above, having fewer. The linguistically annotated version of each corpus adds linguistic annotation, and is distinguished from the “plain-text” one by its files having the .ana suffix, e.g. ParlaMint-XX.ana.xml.

### The corpus header

**Fig. 1:**
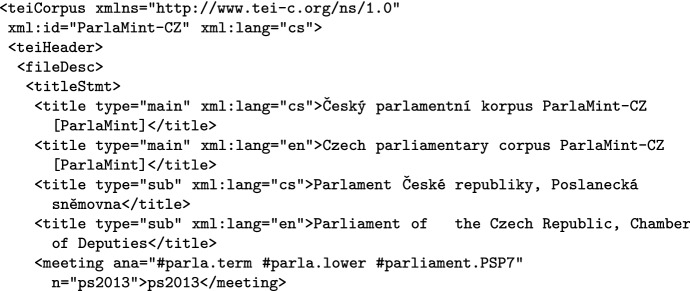
Encoding of the start of a corpus header

Each corpus header has an identical structure across ParlaMint, and encodes considerable information about the corpus, using 78 different TEI elements. In Fig. 1 we illustrate the encoding by giving the start of the Czech corpus header, which sets its ID and language, gives the main boiler-plate title and partner-chosen subtitle and, via the meeting element(s), the term(s) that the corpus covers. It should be noted that we give the titles both in English and in the language of the corpus, and this principle is followed in most of the metadata, so that researchers interested in their country’s corpus have access to metadata in their own language, as well as understanding the metadata of the other corpora.

**Fig. 2:**
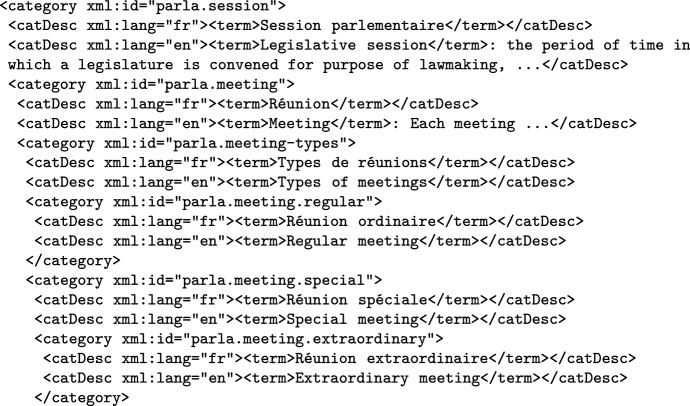
Example of encoding of a legislature taxonomy category

The corpus headers also have a number of controlled values encoded in TEI taxonomy elements, which give IDs to their categories, so they can be referred to from the appropriate elements. One of the more important taxonomies is for legislature, which was initially made for the Slovenian corpus, and then copied and modified as necessary for the other corpora; a category from this taxonomy from the French corpus is given in Fig. 2. The linguistically annotated version of the corpora also contains in their corpus headers taxonomies encoding the set of syntactic relations and NE categories used in the corpus.

**Fig. 3:**
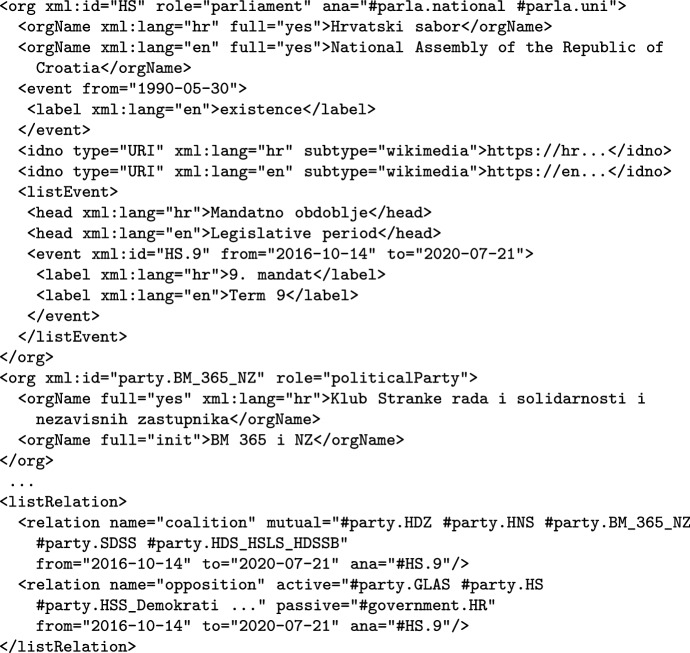
Example of parliament, political party and coalition/opposition encoding

The corpus headers also encode the political parties and other “organisations” that play a role in the parliament, as illustrated in Fig. 3 for the case of Croatian. Each organisation is given an ID so that it can be referred to. Note that the parliament organisation also specifies its legislative periods that are relevant for ParlaMint. The example also illustrates the encoding of coalitions and oppositions, with the first one being a mutual relation between parties, and the second an active relation between the government and the opposition parties.

**Fig. 4:**
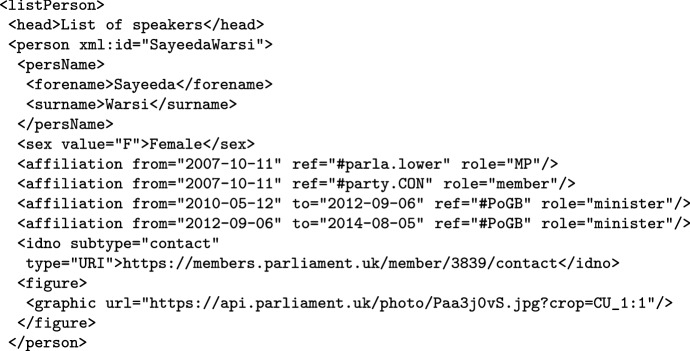
Example of a speaker encoding

The corpus header furthermore contains the list of speakers, as illustrated in Fig. 4 for a speaker from the Great Britain corpus. As mentioned, certain features of the speakers are (near) universal in the ParlaMint corpora, while others, such as various URLs and pictures, are given only for some of them.

The corpus header contains other information as well, e.g. edition, funding and licensing information, the size of the corpus, its tag usage, description of the corpus source and encoding etc. The linguistically annotated version additionally includes in the appInfo element a list of the tools used to annotate the corpus.

### The component header

**Fig. 5:**
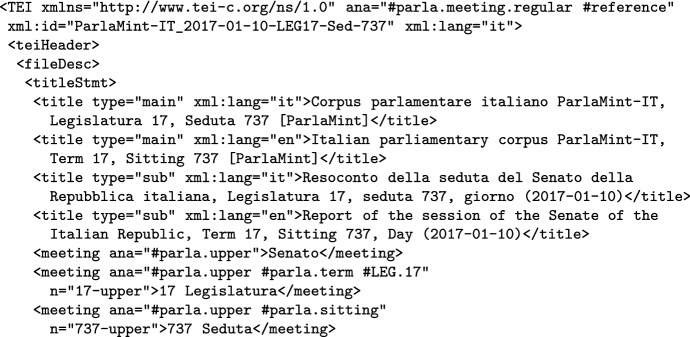
Example of encoding the start of a corpus component header

As mentioned, each corpus component is stored in a separate file, and is rooted in the TEI element, which has its own TEI header followed by the text of the transcription. The component header mostly contains only information specific to its transcription, in particular the corpus-wide unique title and ID, and where in the meeting-type taxonomy the component is placed, also giving the number of the term, session, etc. as is illustrated in Fig. 5 with the start of an Italian corpus component.

### The speeches

**Fig. 6:**
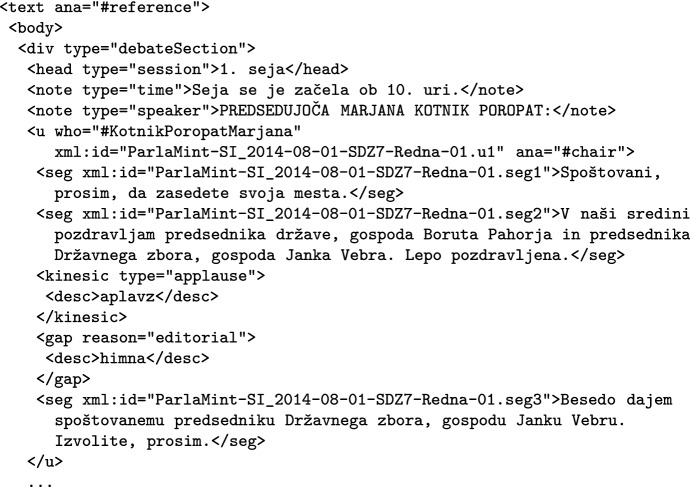
Example of encoded text with speeches

The transcription of a component is, as mandated by Parla-CLARIN, contained in one or more debate sections, which can start with typed headings, and/or notes, as illustrated in Fig. 6 on the case of a Slovene component text.

The main content of the transcription are, of course, the speeches, encoded as u (utterance) elements, which are given references to speaker IDs, and marked for the type of speaker, in particular chair, regular or guest. The speech itself is then encoded as segments, which correspond to paragraphs in the transcription. At any level (so, between speeches, between or inside segments), there can also be notes or incident-encoding elements. Gaps are also allowed, when a part of the transcription was omitted for editorial reasons, e.g. the words of the national anthem. As already mentioned, the amount of detail in the notes and incident elements varies between the corpora, and the values of the type attribute have not (yet) been harmonised between the corpora.

### Linguistic mark-up

**Fig. 7:**
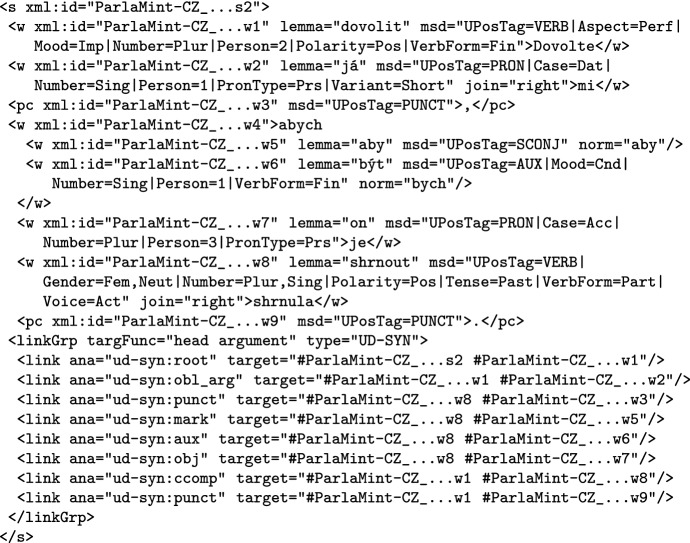
Example of a linguistically analysed text

In the linguistically annotated variant, the textual contents of the segments are marked up with sentences and tokens (words or punctuation marks), which are also given linguistic attributes and a syntactic analysis, as shown in Fig. 7 for a sentence from the Czech corpus. In particular, the lemma attribute of words contains their base form, the msd attribute of tokens contains the Universal Dependencies part-of-speech and morphological features, while the dependency relations are encoded in a stand-off manner in the linkGrp element. If a token is not followed by a space in the transcription, this is indicated by the join attribute. It should be noted that the Universal Dependencies formalism allows so-called syntactic words, i.e. encoding cases where one surface word corresponds to two or more syntactic words as is often the case with clitics. In ParlaMint, syntactic words are encoded as nested words which do not have content but do use the norm attribute, which gives the “normalised” syntactic word, as illustrated by the fourth word in Fig. 7, where the surface word “*abych*” is decomposed into the syntactic words “*aby*” and “*bych*”, and these are then given their linguistic analysis.

The example does not show the annotation of named entities, but this is rather simple, with the tokens constituting it being contained in the name element, with its type attribute giving the type of entity.

## Corpus distribution

One of the goals of the ParlaMint project was to make the produced corpora as openly and FAIRly available as possible. In this section, we present the ways in which the ParlaMint corpora are accessible: through on-line analysis tools, on the CLARIN.SI repository, and via GitHub.

### On-line analysis

In order to make the ParlaMint corpora immediately useful for researchers from the digital humanities and social sciences the corpora were converted and mounted on several on-line analysis platforms.

The Slovenian CLARIN.SI maintains two concordancers, KonText^64^ (Machálek, 2020) and noSketch Engine^65^ (Kilgarriff et al., 2014). They support meta-data based subcorpus creation, configurable concordances, frequency, keyword and collocation lists, and a RESTful interface and API. The two concordancers share the Manatee (Rychlý, 2007) back-end, which enables complex queries over large and richly annotated corpora.

An XSLT stylesheet was used to convert the ParlaMint corpora to the so-called vertical format as used by the concordancers, which is a combination of XML-like structural tags and tabular (TSV) encoded tokens with their annotations. Apart from format conversion, the stylesheet also flattens the corpus text structure to just speeches, which, however, have a full set of attributes, such as the political affiliation of the speaker, their MP status etc. on the date when (s)he is speaking. This transformation makes it easier to use the corpus in the concordancers, as hierarchical structures are more difficult to use properly.

In addition to the concordancers, ParlaMint corpora were also mounted on the ParlaMeter platform,^66^ developed by the Slovenian NGO “Today is a new day”^67^ for journalists and the general public in Slovenia, Croatia, Bosnia and Poland to gain insights into the workings of their parliaments. For example, it gives the most salient keywords of an MP, their vocabulary richness, in which sessions they spoke and what, etc.

### Corpus download

The ParlaMint corpora can be downloaded from the Core Trust Seal and CLARIN certified CLARIN.SI repository,^68^ where each deposited resource has a landing page with the metadata of the resource, the way in which it should be cited, the bit-streams (i.e. downloadable files) associated with it, and links to the corpora on the two concordancers.

The text and linguistically annotated ParlaMint resources (Erjavec et al., 2021, 2021) have one compressed file for each country corpus, and an archive copy of the GitHub repository that corresponds to the resource release. One corpus file unpacks into the source ParlaMint XML files as well as several derived formats:

- Per-speech full metadata (19 columns) TSV files;
- Plain text files, each line marked with speech ID;
- CoNLL-U files, which also include NE annotations in IOB format;
- Vertical files as used by the concordancers including the registry files, so they can be indexed and mounted on any other noSketch Engine installation, on the commercial Sketch Engine,^69^ which supports more advanced features for corpus exploration, or (with some small changes) on any CWB-type (Evert and Hardie, 2011) concordancer.

### ParlaMint on GitHub

As mentioned in Sect.  2.4, a large part of the project development proceeded via the ParlaMint repository on GitHub,^70^ which contains samples of all the corpora, including the derived formats, as well as the root and component TEI headers containing the complete corpus metadata, including the tag counts of the full corpora, making them useful for statistic analyses of the corpora. A script is also used to generate a TEI header encompassing all the ParlaMint corpora, which gives a synthetic view of the complete dataset.

The Git repository also includes all the (XSLT and Perl) scripts used to validate, finalise, and transform the ParlaMint corpora into the derived formats and to compute various statistics, e.g. the Tables in this article. Apart from using them on new corpora, the scripts are also useful as examples of how to transform the corpus into further derived formats for use with other analysis tools. Namely, the ParlaMint TEI encoding makes significant use of pointers and date-stamped elements, and it is useful to see how such information is resolved to arrive at per-speech metadata.

Finally, the GitHub repository also contains open issues where problems with the current set of corpora have been identified and described, and should be fixed in further releases of the data.

## Conclusions

The paper presented the ParlaMint corpora, including their compilation, quantitative data, encoding and distribution. The corpora have already been used in several studies, most prominently version 2.0 of the resources in the 2021 Helsinki Digital Humanities Hackathon. There, the research questions of the team dealing with the ParlaMint task focused on the identification of differences and similarities in parliamentary debates on the COVID pandemic across Italy, Poland, Slovenia and Great Britain, and a blog post has been published on the results (Calabretta et al., 2021). The results of the ParlaMint project were also presented at a CLARIN Café^71^ in June 2021.

As regards future plans, we will continue our work on the ParlaMint corpora in a follow-up project. First, in order to successfully extend the corpora, the documentation and validation of the ParlaMint format should be improved. Currently, the Parla-CLARIN recommendations are lagging behind ParlaMint, even taking into account that Parla-CLARIN is meant to be more general and accommodate corpora with different information than ParlaMint. Conversely, ParlaMint lacks proper documentation on exactly how the corpora are to be encoded, with the GitHub examples currently serving instead of documentation. This means that both should be improved before attempting further ParlaMint extensions. Apart from the validation against the schemas, the XSLT validation currently needs to be run either by the authors of each corpus, or run centrally by the corpus editor. We plan to integrate the validation directly into GitHub as continuous integration.

Second, we plan to extend the corpora both with new countries (and regions) and with new, more recent data for the existing corpora. Here we also plan to add further useful metadata to the corpora, e.g. info on the members of the government (ministers), and grouping of political parties into left, centre and right-wing. We would also like to establish connections with other on-going projects with similar goals, in particular, the EU NORFACE partnership programme “Democratic governance in a turbulent age” project EUINACTION “Willingness and Capacity for EU Policy Action in Times of Crises: Conflicts, Positions and Outcomes”^72^ which plans to compile a corpus of parliamentary debates of all EU countries.

Third, we would like to enrich the ParlaMint data in two directions. The first is machine translating all the corpora to English, most likely using OpenNMT (Klein et al., 2017) with which we have already experimented on ParlaMint corpora with surprisingly good results for most languages. Having all the corpora in English would enable a whole new dimension of comparative studies. The second direction is adding and aligning speech data to the transcriptions; given the complexity of this task, and the fact that not all parliaments distribute their audio, this will most likely be performed only for a few corpora, and is essentially meant as a proof-of-concept study to test and develop the workflow.

Fourth, we intend to work on engagement activities. We will develop a tutorial for digital humanities and social sciences students and scholars, which will demonstrate the use of ParlaMint corpora using a user-friendly text mining tool. To address a very different but important community of users and expose the created resources to novel approaches, we plan to organise a shared task in which the ParlaMint corpora will be used to e.g. predict party affiliation and political ideologies. Finally, we would also like to collect showcases from the ParlaMint community that demonstrate the use of the developed resources to answer research questions from history and political studies.

## Footnotes

1 https://www.ipu.org/parliaments-in-time-pandemic.

2 http://www.talkofeurope.eu/.

3 https://www.clarin.eu/event/2017/clarin-plus-workshop-working-parliamentary-records.

4 https://www.clarin.eu/resource-families/parliamentary-corpora.

5 https://www.clarin.eu/event/2019/parlaformat-workshop.

6 https://clarin-eric.github.io/parla-clarin/.

7 See also Truan and Romary (2020) for a similar but less extensive proposal.

8 Also resulting in a new and bilingual English-Slovene version of the tutorial (Fišer and Pahor, 2021).

9 https://www.clarin.eu/content/parlamint-towards-comparable-parliamentary-corpora.

10 https://github.com/clarin-eric/ParlaMint/.

11 It would be ideal for the complete corpora to be also available from GitHub, however, their large size precludes using Git for this purpose.

12 https://www2.helsinki.fi/en/helsinki-centre-for-digital-humanities/helsinki-digital-humanities-hackathon-2021-dhh21.

13 https://www.dekamer.be/.

14 https://pypi.org/project/langdetect/.

15 http://political.webclark.org.

16 https://www.parliament.bg/en/plenaryst.

17 https://pypi.org/project/classla/.

18 https://stanfordnlp.github.io/stanza/.

19 We follow the evaluation from the CoNLL 2018 shared task, https://universaldependencies.org/conll18/evaluation.html.

20 https://parlametar.hr/.

21 https://lindat.mff.cuni.cz/services/nametag/.

22 https://ufal.mff.cuni.cz/nametag/2/models#czech-cnec2.

23 https://lindat.mff.cuni.cz/services/udpipe/.

24 https://ufal.mff.cuni.cz/udpipe/2/models#universal_dependencies_26_models.

25 ftp://oda.ft.dk.

26 http://hdl.handle.net/20.500.12115/8.

27 https://clarin.dk/clarindk/tools-texton.jsp.

28 https://github.com/ITUnlp/dapipe.

29 The F1-score figures for lemmatisation and name entities annotations are calculated excluding errors following an error in the morphosyntactic annotation.

30 https://data.assemblee-nationale.fr/travaux-parlementaires/debats.

31 https://data.assemblee-nationale.fr/acteurs/.

32 https://www.parlament.hu/.

33 https://rgai.inf.u-szeged.hu/magyarlanc.

34 https://rgai.inf.u-szeged.hu/node/109.

35 https://www.althingi.is/.

36 https://github.com/mideind/Tokenizer.

37 https://github.com/bennigeir/NER.

38 https://dati.senato.it.

39 https://www.saeima.lv/.

40 http://nlp.ailab.lv/.

41 https://zenodo.org/communities/polafra.

42 https://e-seimas.lrs.lt/portal/documentSearch/lt.

43 https://www.lrs.lt/sip/portal.show?p_r=35391&p_k=1.

44 https://www.lrs.lt.

45 https://spacy.io. Information on the general accuracy of the tool (v 3.0.0) for Lithuanian language is provided here: https://github.com/explosion/spacy-models/releases/tag/lt_core_news_lg-3.0.0.

46 https://www.officielebekendmakingen.nl.

47 http://clip.ipipan.waw.pl/PPC.

48 https://sejm.gov.pl, https://www.senat.gov.pl.

49 http://morfeusz.sgjp.pl/.

50 http://zil.ipipan.waw.pl/Concraft.

51 http://nlp.pwr.wroc.pl/narzedzia-i-zasoby/narzedzia/liner2

52 http://zil.ipipan.waw.pl/PDB/PDBparser

53 https://www.congreso.es.

54 The archive is available at http://ecpc.xtrad.uji.es/glossa/html/index.php?corpus=ecpc and at http://150.128.97.136/CQPweb/.

55 https://stanfordnlp.github.io/stanza/.

56 https://www.tbmm.gov.tr/Tutanaklar/TutanakMetinleri.

57 https://github.com/coltekin/ParlaMint-TR.

58 https://github.com/snnclsr/ner.

59 https://hansard-api.parliament.uk/.

60 https://www.api.gov.uk/.

61 https://stanfordnlp.github.io/CoreNLP/.

62 In the corpus we distinguish political parties from groups of parties forming a common list in the parliament. In Table 2 we count both as “Parties”.

63 It should be noted that we here count all the defined persons in the corpora, regardless of how many speeches—if any—they have.

64 https://www.clarin.si/kontext/.

65 https://www.clarin.si/noske/.

66 https://parlamint.parlameter.org/.

67 https://danesjenovdan.si/en.

68 http://clarin.si/repository/xmlui.

69 https://www.sketchengine.eu/.

70 https://github.com/clarin-eric/ParlaMint/.

71 https://dighumlab.org/events/clarin-cafe-parlamint-unleashed-28-june-2021-virtual/.

72 https://www.norface.net/project/euinaction/.
